# Clinical empathy in medical students in India measured using the Jefferson Scale of Empathy–Student Version

**DOI:** 10.3352/jeehp.2017.14.33

**Published:** 2017-12-27

**Authors:** Anirban Chatterjee, Rajkrishna Ravikumar, Satendra Singh, Pranjal Singh Chauhan, Manu Goel

**Affiliations:** 1 Department of Community Medicine, UCMS and GTB Hospital, Dilshad Garden, Delhi, India; 2 Department of Physiology, UCMS and GTB Hospital, UCMS and GTB Hospital, Delhi, India; 3 Undergraduate Student, UCMS and GTB Hospital, Delhi, India; Hallym University, Korea

**Keywords:** Observational study, Medical students, Empathy, India, Surveys and questionnaires

## Abstract

**Purpose:**

The purpose of this study was to assess the clinical empathy of a cohort of medical students spanning 4 years of undergraduate study and to identify factors associated with empathy.

**Methods:**

A cross-sectional study to assess the empathy of undergraduate medical students at the University College of Medical Sciences and GTB Hospital in Delhi, India, was conducted using the Jefferson Scale of Empathy–Student Version. Demographic data were obtained using a pre-tested, semi-open-ended questionnaire.

**Results:**

Of the 600 students, 418 participated in the survey (69.7%). The mean empathy score was 96.01 (of a maximum of 140), with a standard deviation of 14.56. The empathy scores decreased from the first to the third semester, plateaued at the fifth semester, and rose again in the seventh semester. Empathy was found to be significantly associated with the gender of the participant, with females having higher scores (P<0.001). The age of the participant, place of residence, whose decision it was for the student to enroll in an MBBS (bachelor of medicine and bachelor of surgery) program, and the choice of future specialty were not significantly associated with students’ empathy scores.

**Conclusion:**

The study found significant gender differences in empathy among the participants. The empathy scores tended to decline initially and then rebound over time. The mean empathy levels found in this study are lower than those reported in most similar studies around the world; therefore, further studies are needed to analyze and address the underlying factors associated with this discrepancy.

## Introduction

Mercer and Reynolds defined clinical empathy as the ability to understand the patient’s situation, perspective, and feelings (and attached meanings), communicate that understanding and check its accuracy, and act on that understanding with the patient in a helpful (therapeutic) way [1]. Clinical empathy is known to increase patients’ sense of satisfaction, thereby facilitating their compliance [2]. Empathetic doctors are therefore found to make better clinical decisions [3] and be more effective at being transformational leaders. Various scales have been developed to measure clinical empathy. The Jefferson Scale of Empathy (JSE) has seen particularly widespread use among medical students, as a tailored version of the JSE (the JSE-S) was developed specifically to gauge clinical empathy in medical students. The JSE-S has high internal consistency, with a Cronbach alpha value of 0.80, and has been used before amongst medical students across the world, thereby generating comparable results from different cultural contexts.

Studies that have explored the link between clinical empathy and progressive years of medical training have yielded mixed results, with some studies indicating a decline in clinical empathy over time [4,5], some showing no change, and some reporting an increase in clinical empathy. It has also been shown that females are, on an average, more empathic than males in their outlook vis-à-vis patients [6,7]. Other factors, such as the choice of specialty [8], also have a bearing on the levels of clinical empathy that a medical student, and by extension, a doctor displays. Only a single study from India has previously assessed clinical empathy explicitly in medical students, with results indicating a poor mean empathy score [4]. The present study aimed to assess clinical empathy and the various associated factors in a cohort of medical students spanning 4 years of undergraduate study.

## Methods

### Study design

A cross-sectional observational study was conducted amongst undergraduate medical students of University College of Medical Sciences and GTB Hospital with a survey tool.

### Materials and subjects

The JSE-S was used to assess clinical empathy in medical undergraduate students. The English version of the questionnaire was used. Data regarding age, semester, gender, whose decision it was for the student to enroll in undergraduate medical training, choice of specialty, and current place of residence were also collected.

The JSE is a self-administered inventory that contains 20 questions, half of which are negatively phrased, while the other half is positively phrased. The students mark 1 of the 7 options provided on a Likert scale in response to each item (1=strongly disagree, 7=strongly agree). This scale is reversed (that is, 1= strongly agree, 7= strongly disagree) for the negatively-phrased items. It is a 3-factor latent variable scale, with the 3 factors being “perspective taking,” “compassionate care,” and “standing in the patient’s shoes.” Permission to use the questionnaire was obtained [9].

Initially developed in 2001, the scale has since been refined and tailored into 3 versions. Since its development, the scale has been validated elsewhere [7,10].

All medical undergraduate students of University College of Medical Sciences and GTB Hospital in 2017, numbering 600 in total, were invited to participate in the study, and data were collected over a period of 1 month (July 2017). The students were approached in their respective lecture halls at the end of lectures, and printed questionnaires were provided that were to be filled out and submitted in the class itself. Students who could not be contacted in the lecture halls were contacted personally. A total of 3 attempts were made to contact each student.

### Technical information

After obtaining ethical clearance from the Institutional Ethics Board, the investigators distributed and collected the completed questionnaires. Before distributing the forms, the investigators explained the purpose of the study and emphasized that anonymity would be maintained throughout the study period. After completing the questionnaire, the participants were instructed to submit it to the investigators.

### Statistical analysis

The data were entered in a computer-based spreadsheet and analyzed using R version 3.4.1 (https://www.r-project.org/). Missing demographic data were coded as missing and excluded from the analysis. The scoring algorithm allowed for a maximum of 4 blank items (out of the 20), in which case the missing values were replaced by the mean score of the items that the participant responded to. If more than 4 items had no response, the form was considered incomplete and excluded from the analysis. Reverse-scored items were scored accordingly.

After conducting the descriptive analysis, the totaled empathy scores were compared according to gender (male or female), semester (first, third, fifth, or seventh), whose decision it was for the student to enroll in the undergraduate medical curriculum (one’s own or that of parents/relatives), choice of specialty (people-oriented, technologyoriented, or others), and current place of residence (home or other). Comparisons according to gender, who decided for the student to enroll in the undergraduate medical curriculum, and current place of residence were conducted using the Student t-test, while those for semester and choice of specialty were conducted using analysis of variance. The Bonferroni post hoc test was used for semester and choice of specialty. A correlation analysis was also performed between mean empathy scores and the age of the participants. A P-value of < 0.05 was considered to indicate statistical significance.

### Ethical approval

Prior to conducting the research, ethics clearance was obtained from the Institutional Review Board of the University College of Medical Sciences and GTB Hospital, New Delhi (vide ref no. IEC-HR/2017/ 31/5) after receiving informed consent from the subjects.

## Results

Of a total of 600 students, 418 participated, representing a participation rate of 69.7%. Of these participants, an overwhelming majority (331, 79.2%) were males, while the rest (87, 20.8%) were females. Information regarding the distribution of males and females across semesters is provided in Table 1. The raw data are available in Supplement 1.

**Fig. 1.**
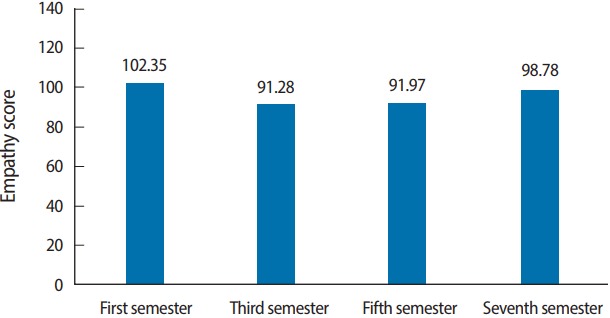
Bar graph depicting the empathy scores by semester.

The mean empathy score in our study was 96.01 out of a maximum of 140, with a standard deviation of 14.56. The 20-item empathy scale was observed to have good internal consistency in this population group (α= 0.765). A dip was observed in the mean empathy scores from the first to the third semester, but then it plateaued and recovered by the seventh semester, as can be seen in Table 2 and Fig. 1. This change was observed irrespective of gender, place of residence, whose decision it was for the student to enroll in the undergraduate medical curriculum, or the choice of specialty (Table 3). Empathy was also found to be significantly associated with gender, with females being more empathetic than males (Table 2). This difference tended to diminish as the semester of study increased, such that by the seventh semester, no significant difference was seen in the mean empathy scores of females and males.

Clinical empathy was not significantly associated with age (r=-0.71, P= 0.153), place of residence, or whose decision it was for the student to enroll in the MBBS (bachelor of medicine and bachelor of surgery) program. The future choice of specialty was grouped into 3 categories (people-oriented, technology-oriented, and others). No significant association was found between clinical empathy and the choice of specialty (P= 0.054) (Table 2).

The 3 factors that make up the scale—compassionate care, perspective taking, and walking in the patient’s shoes—were also analyzed, and their means with standard deviations are given in Table 4.

We also compared the mean empathy scores observed in our study with those from studies elsewhere on the Indian subcontinent, as well as from other countries. These findings are presented in Table 5. As can be seen in Table 5, the empathy scores observed in our study are amongst the lowest that have been recorded.

Post hoc tests indicated that there was a significant difference in the mean empathy scores from the first to the third semester (P<0.001), and from the fifth to the seventh semester (P= 0.003), but not between the third and the fifth semester (P> 0.999). No significant difference was found according to the 3 categories of the choice of specialty in post hoc testing.

## Discussion

The aim of this study was to assess clinical empathy in medical undergraduate students and to identify factors associated with empathy. The outcomes of this study can be discussed under the following broad sub-headings.

### Clinical empathy and gender

In our study, clinical empathy was found to be significantly associated with gender, with females having significantly higher mean empathy scores than males. This difference tended to diminish over the semesters, such that by the seventh semester, no significant difference was seen in the mean empathy scores of female and male participants. Since this is a cross-sectional study, the temporal significance of this finding cannot be definitively explained, but similar findings have not been reported elsewhere. In a study in Pune, it was found that there was no significant change in the mean empathy score of females across the semesters, but males showed a decline [4]. A longitudinal study conducted by Hojat et al. [11] using the same questionnaire found that although mean empathy scores in males and females changed equally over the years, females showed consistently higher scores than males, even when the mean scores dipped in general, and that the difference remained significant. Further studies are warranted to explore the temporal trend of clinical empathy in both male and female medical students.

Many studies have shown that the mean empathy scores of female medical students were higher than that of males, including studies carried out in Pune and Bangladesh [4,7]. The most common explanation for this finding has been said to be the expectations associated with traditional gender roles, but a study conducted by Baez et al. [12] in 2017 found that tools that rely on self-reporting for estimating empathy may induce biases leading the participating individual to assume traditional gender-based stereotypes. In contrast, a review conducted by Christov-Moore et al. [13] asserted that higher empathy in females has not only social, but also phylogenetic and ontogenetic roots. A few studies have also found no differences in the mean empathy scores of female and male medical students [10]. Further studies are therefore needed to explore the associations of sex and gender roles with clinical empathy.

### Clinical empathy and number of years of study

In our study, the mean empathy scores fell from the first to the third semester, then more or less plateaued, and then rose again in the seventh semester. Associations of the number of years of medical education with empathy scores have been explored in many other studies, of which a few have found an increase in clinical empathy with increasing number of years of education [14], some have found a decreasing trend over the years [4,7], and others have found no significant difference in empathy scores across the years of medical education [10]. The findings of our study were unique, in the sense that clinical empathy was seen to increase after the fifth semester in students in the seventh semester. Since this was a cross-sectional study, it is difficult to draw any temporal inferences, but a similar finding in another study from India [4] indicates that further studies should be conducted to explore this phenomenon and its possible causes. It may be the case that a higher empathy score in the seventh semester indicates the positive effects of community medicine (previously called social and preventive medicine) being taught in the sixth and seventh semesters. It has been found elsewhere that doctors of family medicine (loosely an off-shoot of community medicine) are more empathetic than others [15].

### Clinical empathy across different settings

In general, the mean empathy score in our study (96.01, standard deviation=14.56) is lower than has been reported in most other studies that have been conducted in Asia [4,7] or in Western countries [11], a finding that is worrisome. It has been asserted that physicians in Asia in general adopt a more paternalistic role in a doctor-patient relationship [16]. This might be partly responsible for explaining our findings, but further investigations are required to identify the factors associated with such low scores, so that steps can be taken to address the situation.

This study has methodological limitations that need to be taken into consideration before interpreting the results. The response rate in our study is a matter of concern; although it is fairly high, non-participants may have been significantly different from those who chose to participate. Our findings must be interpreted in light of this possibility. In addition, self-reporting questionnaires come with their own set of biases, which may have an impact on the results. Setting aside the various reasons the participants might have to underestimate or overestimate their empathy, social desirability may lead them to underreport or overreport empathy. Finally, like all cross-sectional studies, our study cannot be used to comment on causal associations.

In conclusion, the study found significant gender differences in the clinical empathy levels of the participants. The empathy scores were observed to decline initially and then recover as the semester of study increased. The mean empathy level found in this study is lower than has been reported in most similar studies across the world, which is a worrisome finding that requires further analysis.

## Figures and Tables

**Table 1.** Empathy score of participants by gender (n = 418)

Semester	Total	Gender	No. of participants	Empathy score	P-value
1	95	Male	75	99.71	0.001
		Female	20	112.25	
3	115	Male	91	89.35	0.008
		Female	24	98.58	
5	93	Male	76	90.43	0.010
		Female	17	98.82	
7	115	Male	89	98.42	0.552
		Female	26	100.04	

**Table 2.** Associations between various independent factors and clinical empathy

Independent factor	No. of participants	Empathy score		P-value
		Mean ± standard deviation	95% confidence interval	
Gender				<0.001
Male	331	94.38±14.45	92.83-95.93	
Female	87	102.21±13.30	99.41-105.01	
Semester				<0.001
First	95	102.35±15.36	99.27-105.43	
Third	115	91.28±15.26	88.50-94.06	
Fifth	93	91.97±12.29	89.46-94.48	
Seventh	115	98.78±12.18	96.55-101.01	
Decision to join				0.628
Own	357	96.22±14.03	94.77-97.67	
Others'	55	97.22±15.42	93.14-101.3	
Choice of specialty				0.054
People	152	98.15±13.58	95.99-100.31	
Tech	177	94.38±13.84	92.34-96.42	
Others	79	96.78±16.14	93.21-100.35	
Place of residence				0.675
Hostel	266	96.55±14.29	94.83-98.27	
Home	147	95.94±13.92	93.69-98.19	

**Table 3.** Clinical empathy across semesters compared to various independent factors

Independent factors	Mean empathy by semester				F-value	P-value
	1	3	5	7		
Gender						
Male	99.71	89.35	90.43	98.42	12.44	<0.001
Female	112.25	98.58	98.82	100.04	5.831	0.001
Choice of specialty						
People	102.6	93.03	94.68	100.90	4.296	0.006
Technology	102.34	90.6	91.34	95.18	7.438	0.000
Others'	102.14	94	88.83	99.92	3.105	0.032
Decision to join						
Own	101.38	92.52	90.87	99.60	12.95	0.000
Others	111.56	89.40	97.67	96.06	4.72	0.006
Place of residence						
Hostel	103.22	91.00	92.56	98.04	10.25	0.000
Home	100.55	93.40	90.52	100.81	4.75	0.003

**Table 4.** Demographic variables associated with mean empathy component scores

Independent factors	Perspective-taking	Compassionate care	Walking in the patient’s shoes
Gender			
Male	50.93 ± 9.90	35.98 ± 7.52	7.47±2.69
Female	54.48 ± 8.90	39.75±7.37	7.98±2.52
Semester			
First	54.93 ± 9.31	39.15±7.76	8.27±2.83
Third	49.10 ± 10.999	34.16±7.66	7.23±2.48
Fifth	50.05 ± 9.31	34.74±6.92	7.17±2.54
Seventh	52.87 ± 8.34	38.24±7.24	7.67±2.69

Values are presented as mean ± standard deviation.

**Table 5.** Comparison of results from previous studies from different countries using the Jefferson Scale of Empathy – Student Version

Study	Country	Mean 士 standard deviation
Our study	India	96.01 ± 14.56
Shashikumar et al. [4] (2014)	India	102.91 ± 19.217
Mostafa et al. [7] (2014)	Bangladesh	110.41 ± 13.59
Rahimi-Madiseh et al. [10] (2010)	Iran	105.1 ± 12.9
Kataoka et al. [14] (2009)	Japan	104.3 ± 13.1
Hojat et al. [11] (2009)	USA	115.0 ± 10.0

## Supplementary materials

Supplement 1. Data files are available from https://doi.org/10.7910/DVN/LOGUCL

Supplement 2. Audio recording of the abstract.
